# Social dimensions impact individual sleep quantity and quality

**DOI:** 10.1038/s41598-023-36762-5

**Published:** 2023-06-15

**Authors:** Sungkyu Park, Assem Zhunis, Marios Constantinides, Luca Maria Aiello, Daniele Quercia, Meeyoung Cha

**Affiliations:** 1 Department of AI Convergence, Kangwon National University, Chuncheon, 24341 Republic of Korea; 2 Data Science Group, Institute for Basic Science, Daejeon, 34126 Republic of Korea; 3 School of Computing, KAIST, Daejeon, 34141 Republic of Korea; 4 Nokia Bell Labs, Cambridge, CB3 0FA UK; 5 IT University, Copenhagen, Denmark; 6 Pioneer Centre for AI, Copenhagen, Denmark; 7 Centre for Urban Science and Progress, King’s College London, London, UK

**Keywords:** Psychology and behaviour, Human behaviour, Quality of life

## Abstract

While sleep positively impacts well-being, health, and productivity, the effects of societal factors on sleep remain underexplored. Here we analyze the sleep of 30,082 individuals across 11 countries using 52 million activity records from wearable devices. Our data are consistent with past studies of gender and age-associated sleep characteristics. However, our analysis of wearable device data uncovers differences in recorded vs. self-reported bedtime and sleep duration. The dataset allowed us to study how country-specific metrics such as GDP and cultural indices relate to sleep in groups and individuals. Our analysis indicates that diverse sleep metrics can be represented by two dimensions: sleep quantity and quality. We find that 55% of the variation in sleep quality, and 63% in sleep quantity, are explained by societal factors. Within a societal boundary, individual sleep experience was modified by factors like exercise. Increased exercise or daily steps were associated with better sleep quality (for example, faster sleep onset and less time awake in bed), especially in countries like the U.S. and Finland. Understanding how social norms relate to sleep will help create strategies and policies that enhance the positive impacts of sleep on health, such as productivity and well-being.

## Introduction

Recent years have seen breakthroughs in research into the biological, cellular, and biochemical mechanisms underlying sleep. The circadian clock modulates sleep based on the timing and levels of natural and artificial light exposure^1^. However, multiple factors influence sleep, including environmental (e.g., sunset and sunrise times), social (e.g., cultural norms), and individual attributes (e.g., age and gender). The human sleep project advocated equipping people with a range of mobile sleep-tracking devices to study their sleep patterns in real-time and on a large scale, referring to the impact of social factors as the next frontier in sleep research^2^. Studying the interplay between sleep and societal factors necessitates large-scale datasets that bridge multiple societies. Recent app-based surveys and sensing technologies have expanded sleep analysis to the population level^3,4^ and even to a global scale^5,6^. However, most previous sleep studies focused on small-scale data^7^.

Past research indicates that different methods lead to discrepancies with actual sleep levels. Indeed, self-reports, commercial accelerometers, actigraphy, and even the gold standard for measuring sleep, polysomnography, have been shown to overestimate or underestimate sleep^6,8–10^. Recent years have seen advances in commercial devices that record individual activity. A study conducted in 2017 found that although wearable devices were less effective in tracking sleep patterns in individuals with sleep disorders, they were still comparable to the gold standard methods used for monitoring normal sleep^11^. A large dataset from Fitbit was analyzed in another study to examine the variations in sleep behaviors among different social groups^12^. The study found significant differences in sleep patterns based on gender and age among people living in Oceania and East Asia. These findings prompted questions about the potential factors, such as genetic or adaptive mechanisms, that contribute to these differences. With these issues in mind, we assessed wearables as a source of data for examining the influence of *culture* on these sleep patterns, leveraging their wide adoption and ability to track various aspects of human physiology.

Here we analyzed sleep and physical activity data from 30,082 individuals who wore the same wearable device brand. Data from individuals representing 19 cities across three continents were examined. The data allowed us to study sleep across multiple societal boundaries at an unprecedented scale. The 52 million activity records and user demographic information recapitulated known influences of age and gender on sleep. However, bedtimes measured using wearables appeared substantially delayed compared to self-reports, suggesting a systematic reduction in actual sleep duration. We found that social effects explain up to 63% of variations in measurements of sleep quantity measures (e.g., sleep duration), and 55% of the variation in sleep quality measures (e.g., sleep efficiency). When we controlled for country effects, our data show that enhanced or poor sleep experiences are explained by controllable factors such as exercise. The effect size, or the balance between societal and individual influence, varied by country. This interplay between social constructs and individual behavioral efforts has not previously been recorded on the scale examined here. Our analysis provides a quantitative basis for designing guidelines and policy interventions that center on sleep for social and welfare programs.

## Results

**Figure 1:**
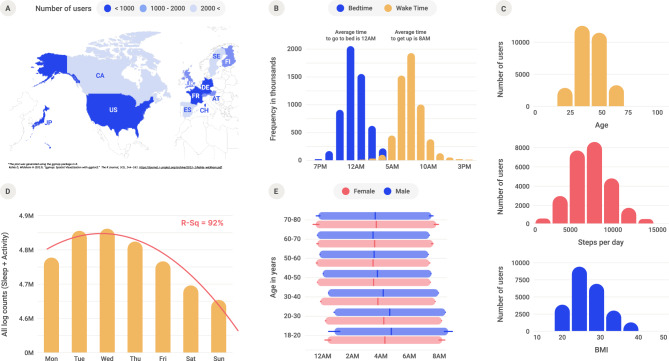
Demographic distribution of study participants. (**A**) Countries included in the study. (**B**) Distribution of bedtime and wake time. (**C**) Distribution of age, daily steps, and BMI among the users. (**D**) Distribution of total data counts by day of the week. (**E**) Sleep scheduling by age and gender (median values with standard error bars).

**Figure 2:**
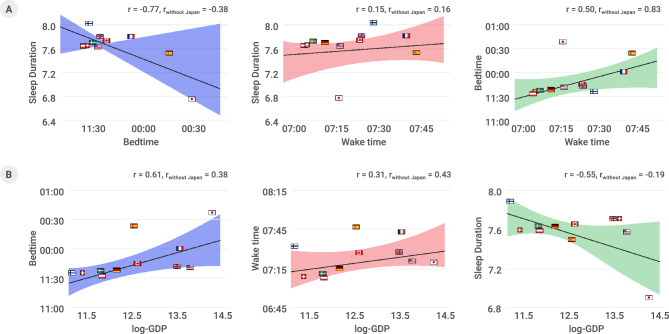
Country effect on sleep duration, indicated by bedtime and wake time. (**A**) Cross-correlation between the median sleep duration, bedtime, and wake time in data from 11 countries. The effect of the residing country on sleep duration primarily manifests through bedtime rather than wake time. (**B**) The effect of log-GDP on sleep, demonstrating that countries with higher GDP exhibit a longer delay in bedtime.

The data analyzed came from 11 different countries (Fig. 1A) and were collected between 2014 and 2017 in 19 cities where the studied wearable device was prevalent. The smallest city contained 495 users, and the largest city contained 8924 users. Sleep was inferred by the sensing technologies in wearable devices and the associated algorithms, which indicated a global median of 12:01 AM bedtime and 7:42 AM wake time, respectively (Fig. 1B). The data contained user demographic information and both daytime and nighttime activity. The user base had a slightly higher presence of males (55%) and older adults (i.e., the median age was 42 years). The median BMI and number of steps per day recorded were 25.4 and 6,951, respectively (Fig. 1C). The day-of-week pattern indicated that, in general, users were most active on Wednesdays (Fig. 1D). This finding is consistent with other observations of higher activity (e.g., trading volume, physical activity) in the middle of the week^13–15^. For all groups younger than 70, bedtimes and wake times were generally much later in females than males (Fig. 1E). Additionally, females had significantly shorter sleep durations than males, particularly for individuals aged 30–40 (refer to Table S6 in Supplementary Material) (Fig. 2).

### Social constructs can explain key sleep measures at the population level

To closely investigate the effects specific to each city, we compiled a total of five literature-driven sleep metrics: sleep duration, sleep history, mid-point in sleep on free days corrected for sleep debt on work days (MSFsc), K-hour deviation, and sleep efficiency (see Methods for more details). After running a principal component analysis (PCA) on these sleep statistics, we identified two prominent dimensions of sleep that represented individual sleep quantity and quality-related measures, respectively. The process of dimension reduction is described in Text S2 of the Supplementary Materials. Together these two dimensions explained 83% of variations in the data, ensuring a good fit.

Testing the presence of country effects on sleep quantity, we also examined how sleep quantity varied according to standard social constructs. In addition to log-GDP, we incorporated Hofstede’s cultural dimensions and considered well-established indices, such as individualism (IDV) and uncertainty avoidance index (UAI), and found meaningful correlations with sleep measures (see Materials and Methods and Fig S2 in Supplementary Material) confirming significant social influence on sleep. In the individual-level assessment, as presented in Table S7 of the Supplementary Material, the city of residence could explain only a marginal proportion of the two dimensions of sleep (Adjusted R^2 0.10 for Sleep Quantity and 0.03 for Sleep Quality). This suggests that the impact on sleep may not be solely determined by location, but rather by the cultural environment of the cities in which individuals reside. Therefore, we examined how the two sleep dimensions are associated with popular social constructs by considering six linear models (M1 to M6) at a city-specific level in Table 1. For each PCA dimension, the baseline models (M1 and M4) only consider log-GDP and control for the median age of users. The full models (M2 and M5) use all social constructs. The final compact models, M3 and M6, are trimmed-down versions that only contain significant factors by the StepAIC feature selection method. The analysis indicates that a few relevant social constructs (such as GDP, IDV, and UAI) alone can explain 63% of variations in sleep quantity and 55% of variations in sleep quality (refer to Table 1). This is strong evidence that both the amount and quality of sleep are associated with the social environment that users experience.

**Table 1:** Regression models between sleep dimensions and social constructs on the city level.

Feature	Sleep quantity (RC1)			Sleep quality (RC2)		
	*M*_1_	*M*_2_	*M*_3_	*M*_4_	*M*_5_	*M*_6_
	Baseline	Full	StepAIC	Baseline	Full	StepAIC
**log-IDV**		0.80**	0.61***		0.62	0.56*
**UAI**		0.14			0.53	0.60*
log-LTO		0.06			0.16	
log-IND		− 0.04			− 0.04	
**log-GDP**	− 0.74***	− 0.90***	− 0.86***	− 0.70***	− 0.86***	− 0.91***
Median Age	− 0.46*	− 0.33	− 0.30	0.04	− 0.02	
*Intercept*	0.00	0.00	0.00	0.00	0.00	0.00
*Adjusted R*^2^	0.29	0.55	**0.63**	0.46	0.46	**0.55**

Statistical significances are marked with the number of *’s based on their significance levels (i.e., ***p < 0:01; **p < 0:05; *p < 0:1)

### Individual activity can improve sleep within societal boundaries

**Figure 3:**
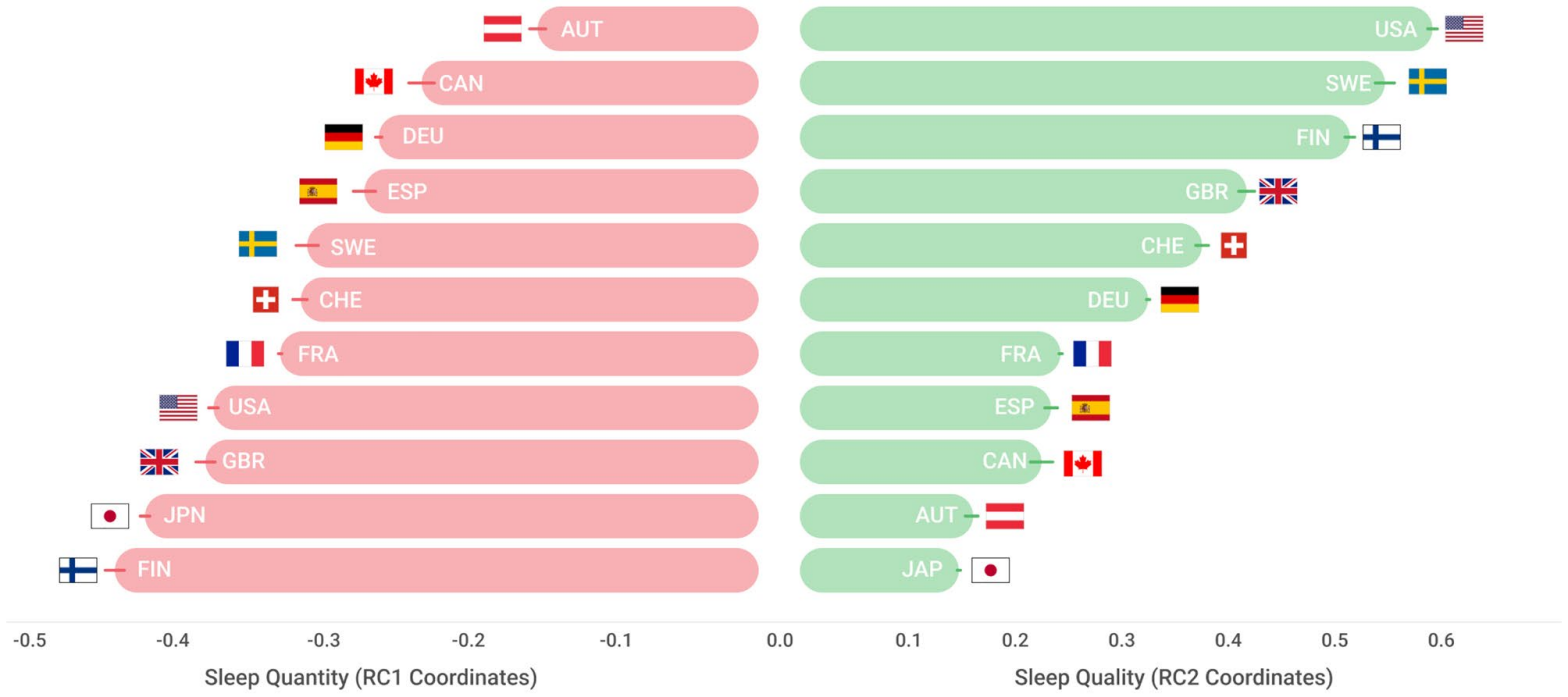
Comparison of sleep between top 30% exercising and non-exercising individuals categorized by country. Propensity matching analysis was utilized to calculate the degree of influence of daily steps on each sleep factor. The x-axis represents the EATE (estimated average treatment effect) of average daily steps on two sleep dimensions: Sleep Quantity (RC1) and Sleep Quality (RC2). Across all target countries, the EATE values were negative for RC1 (indicating less sleep quantity) and positive for RC2 (indicating better sleep quality) for the active group (top 30% of users in terms of average daily steps) compared to the non-active group (bottom 30% of users). This holds across countries but is prominent in, for example, the US and Finland as they are at the bottom of the quantity graph and the top of the quality graph.

In order to uncover individual influences on sleep, we divided the individuals residing in each city into the top one-third and the bottom one-third based on their activity levels (measured by daily steps, as described in Materials and Methods). We then compared their composite sleep dimensions using propensity score matching. We found that individuals who exercise have better sleep efficiency, which means they spend a larger portion of their time in bed actually sleeping. Their total sleep duration is also shorter than that of non-exercising individuals by a small margin. As a result, Fig. 3 shows that exercising individuals exhibit negative sleep quantities compared to less active individuals in the same city, but they enjoy better sleep quality, yet this effect varied depending on the residing country.

To interpret these findings, we ran regression models to find the relationship between sleep and a unit increase in physical activity (refer to Table S8 in Supplementary Material). For instance, among U.S. residents who sleep for 8 h, an additional 1000 steps per day, on average, led to an increase in active sleep duration by 1.28 minutes. However, the total duration spent in bed (sleeping or not) decreased by 2.47 min, perhaps to accommodate the extra time devoted to physical activity. The Supplementary Material contains additional information on relationships between sleep and physical activity, age, and gender relative to cultural dimensions.

## Discussion

In this study, we utilized data from a single brand of wearable devices to assess sleep patterns in a large-scale sample of 30,082 individuals across 11 countries. These findings are discussed further below.

### Data from wearables concur with previously known sleep patterns but reveal substantially delayed bedtimes and shorter sleep duration

While our data confirmed previously studied relationships between bedtime, wake time, and sleep duration (Fig. 2A), the scale and international nature of the data also revealed new insights into sleep patterns. Sleep duration measured using wearable data is more strongly determined by bedtime than wake time, suggesting the influence of social obligations at night time, as seen in survey-based data in previous studies^5^. Multiple epidemiological and cohort studies have shown that increasing age, in general, is associated with shorter sleep duration and earlier wake times^16^. A reduction in total sleep duration of 0.5 min per year of age increase was seen in the large multicenter community Sleep Heart Health research (n = 2113)^17^. Another study revealed that a short sleep duration prevails in teenagers and those aged > 65 years^7^. Our data also show that age has a nonlinear relationship with sleep timing and duration (Fig. S1). The consistency of findings relative to other methodologies indicates that wearable data serve as a valuable comparative tool for sleep studies, as first demonstrated in^11^. Moreover, the wearable data also provide objective, quantitative data measurements.

**Figure 4:**
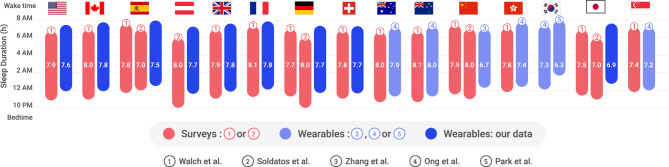
Wearable vs. survey-based studies. A comparison of average bedtime, wake time, and sleep duration based on data results obtained from six different sources across 15 countries^4,5,12,18,19^.

Indeed, the wearable data indicated several visible differences in sleep patterns compared to studies based on self-reporting. For example, previous research found that women sleep more than men and generally have earlier bedtimes and later wake times until the age of 60^5,6^. Meanwhile, our data shows the same patterns for bedtimes, but not for wake times, in female and male cohorts (Fig. 1E). Previous wearable studies also showed that women tend to self-report shorter and less efficient sleep in surveys compared to actigraphy measurements^6^. Our wearable data also reported later bedtimes and wake times compared to survey-based studies in all countries that were examined using both approaches (see Fig. 4). The highest quantity of sleep among the 11 countries, according to wearable data, was found in Finland, where the median daily sleep time was 8 h. The reported sleep time in all other nations was 16–69 min shorter than it was in Finland (Table S1). Data from Japan showed the lowest median sleep duration, 6 h and 51 min, and the biggest difference between wearable-recorded and self-reported sleep measurements.

Focusing on country-specific variations in Fig. 2B, we identified that three sleep measures substantially correlate with log-GDP for the studied countries, a relationship that has not been extensively examined in previous studies. Specifically, bedtime was found to be delayed in higher-income countries, yet wake time was affected to a lesser degree. Higher-income countries also showed shorter average sleep durations. The relationship between GDP and sleep measures observed in our study could be driven by various mechanisms. One possibility is that higher-income countries may have more demanding work schedules or longer working hours, leading to delayed bedtimes and shorter sleep durations^20^. Another potential factor is that higher-income countries may have greater access to technology and entertainment, resulting in later bedtimes and reduced sleep durations due to increased exposure to screens and devices^21^.

According to the analysis, a handful of important social factors such as GDP, Individualism, and Uncertainty Avoidance are able to account for 63% of the differences in sleep quantity and 55% of the differences in sleep quality across different locations (refer to Table 1). This finding suggests that the amount and quality of sleep may be linked to the specific societal and cultural characteristics of the region where individuals reside. For example, considering the baseline models, living in higher GDP regions was associated with shorter sleep duration (*p* < 0.01) and lower sleep efficiency (*p* < 0.01). In contrast, considering the final StepAIC model, countries with higher individualism Index (IDV), which indicates the degree to which a culture reinforces individual achievement and relationships, had longer sleep duration (*p* < 0:01), which may be explained by an earlier bedtime schedule in such societies. Countries with high collectivism scores, i.e., low IDV values, may have stronger social obligations at night, potentially leading to shorter sleep duration. For instance, the cultural emphasis on social harmony and group obligations in collectivistic cultures may create stronger expectations for social engagement or activities at night, which can in turn impact individuals' sleep behaviors. Individuals who work long hours or have rigid work schedules that extend into the evening may also have fewer opportunities for restful sleep. Spain and Japan were the two countries in the studied data that had the highest collectivism scores and the most delayed bedtime schedules (refer to Fig. S2 in Supplementary Material). In contrast, wake times were less divergent across the studied countries. Both high individualism (IDV) index and high uncertainty avoidance (UAI) index, which capture how regulated life is, were also associated with better sleep quality, but to a moderate degree (*p* < 0:1).

Previous survey-based studies have shown that bedtimes and wake times are strongly dependent on demographics such as age and gender. We confirmed the same correlations in Fig. S1. In addition, we newly explored whether these trends can be modified by individually configurable factors. One study conducted a sleep intervention experiment in a poor urban area and showed that half-hour naps at the workplace can induce high-quality sleep and therefore increase work productivity by an average of 2.3% throughout the day^22^. Our analysis of individual factors also highlights, within societal boundaries, that increasing daily steps can further affect sleep (see Fig. 3). People who exercised had better sleep quality, and their total sleep duration was shorter than that of less active individuals. The effect of exercising and increased daily activity was more pronounced in some countries (e.g., the U.S., Finland) than in other countries (e.g., Austria). We also found that Japan was an exceptional case where additional daily steps did not lead to increased sleep quality. These findings suggest that the relationship between daily activity and sleep may be country-specific, and different exercise regimes may be more effective for some cultures than others. Further research could explore this possibility in more depth.

### Theoretical implications

Our findings have three theoretical implications. First, we provide evidence that societal influences should be considered in modeling sleep. Previous literature showed physiological and environmental effects on sleep and suggested that individuals have unique internal time signatures^23^. Along with recent literature that discusses social influences^2,5,6^, our work offers strong evidence, supported with extensive data, that social constructs can explain the majority of variations in sleep patterns (Table 1).

The second theoretical implication is that our results bring clarity and structure to the numerous sleep metrics available in the literature^24–27^. Upon analyzing seven metrics, we found that sleep patterns can largely be summarized using two orthogonal dimensions (83.14% of the explained variability): Principal Component Analysis (PCA), based on our data, shows that sleep metrics can be classified into those reflecting quantity (i.e., sleep duration, sleep history, and MSFsc), and those reflecting quality (i.e., sleep efficiency, the K-hour deviation). These metrics were chosen based on their widespread use in sleep research and their relevance to capturing important aspects of sleep patterns.

The third theoretical implication comes from the limitations of existing surveys on sleep. Many studies have captured intended or desirable sleep behaviors, but very few have examined sleep in relation to participants' other life signals. This limitation can be rectified by ensuring that surveys contain questions that give insight into activities related to well-being^28,29^. Surveys can include questions about expected work shift timing, the number of holidays, access to electronic devices in the bedroom, and cultural expectations about sleep. Our results suggest a tradeoff between one’s social life and sleep patterns. Societies that sacrifice a bit of sleep tend to show markers of better social life; they focus on the collective experience (Collectivism, i.e., smaller log-IDV values) by enjoying the moment and spontaneous lives (i.e., smaller Uncertainty Avoidance Index values). We also saw a poorer sleep tendency, both in terms of quantity and quality, in cities with higher GDP (i.e., larger social scales).

### Practical implications

Our findings also have two practical implications. The first concerns multinational organizations. Based on our findings, international organizations may want to vary non-work activities and tailor them to local societal norms. For example, some societies emphasize individual work over sleep. This tendency could be counterbalanced by generous after-work activities that support productivity and, ultimately, well-being.

The second practical implication concerns sleep-tracking apps. These apps should consider not only “who the user is” (i.e., individual characteristics, including exercise, diet, nutrition, and quality of life) but also “where (s)he lives” (i.e., social influences and scales). For instance, there is a strong correlation between economic indicators like GDP and the quantity of artificial light present at night^30^. Since excessive exposure to artificial light may impact sleep and health, app designers may want to take these local factors into account in future iterations of their sleep devices^31^. By implementing this information, an app could recommend optimal sleep patterns for users that, for example, do not compromise an individual’s social life.

### Limitations and future work

This work comes with several limitations. One is measurement accuracy in sensing sleep. Although wearable devices are advancing and are widely used, the gold standard for measuring activity signals consists of less ubiquitous actigraph sensors or polysomnography^32^. Such direct measurement methods can detect variant sleep patterns. Other less invasive methods, like survey-based research, suffer from reporting biases. In fact, our data show a consistent deviation between sleep data obtained from surveys and wearable devices. Figure 4 shows average sleep schedules obtained from six different sources across 15 countries (see Table S3 for more detailed statistics), where bars indicate time frames when individuals went to bed and woke up on average. In the surveys, users reported going to bed earlier at night and waking up earlier in the morning compared to users in wearable-based studies across multiple data sources. To increase accuracy, researchers can make existing wearables ‘smarter’ with on-device machine learning^33^ or make existing high-precision devices less invasive by miniaturizing them^34^. Furthermore, deep-learning algorithms could be used to fill in missing data points and prune possible outliers^24^.

Another limitation concerns the control variables and generalizability. While our study controlled for two common factors directly related to sleep (i.e., overall socioeconomic status of a city and median age), future studies could control for additional variables (e.g., gender or income) whenever available. Previous research emphasized the potential for interaction between, as well as the mutual influence of, genetics and culture^35^. As noted by Ong et al.^12^ it is also plausible that genetic variation accounts for cultural disparities in the sleep habits of users. For example, it has been hypothesized that East Asians may have genetic variations that give them resilience to sleep deprivation^36^. By examining how genetic information affects these disparities, future research may reveal the mechanisms underlying differences in sleep patterns across ethnic groups.

Also, our findings are limited to countries with relatively higher GDP values (i.e., at least $252 billion or within the top 42 countries as of 2023) and may not be generalizable to more socio-economically deprived countries. Furthermore, tech-savvy individuals will tend to wear high-end tracking devices, leading to potential population bias in wearable-based studies. Despite this potential homogeneity in our sample, there were still stark differences in sleep quality that were explained by social constructs.

### Ethical considerations

This study adhered to the General Data Protection Regulation (GDPR) in terms of data collection, processing, and storage. The authors had no access to personally identifiable information of individuals in the studied dataset. All data were analyzed at the aggregate level (i.e., cities, countries, and groups by sleep patterns).

## Materials and methods

### Data collection

As consumer-grade wearables are now fully equipped with body sensors, it is possible to gather measures related to an individual’s well-being (e.g., physical activity or sleep) at a large scale^24,25^. We obtained sleep readings from commercial wearable trackers worn by 30,082 unique users (55% male and 42 years old median age with a standard deviation of 12 years) between 2014 and 2017 across 19 major cities and capitals in the countries with GDP per capita rank above 34 and GDP above 42 [https://www.worldometers.info/gdp/gdp-by-country/], spanning three continents. The city with the lowest penetration had 495 users (median = 1043 and mean = 1588). In total, we collected 28.5 million sleep measurements. The device inferred sleep based on a combination of sensors and reported the measurements as presented in Table 2.

**Table 2:** Descriptive statistics of the sleep readings obtained from wearable devices.

Aggregated as the average by city				
Variable	Meaning	Median	Mean	SD
Bedtime (unit: timestamp)	The estimated time at which a user gets into bed for sleep	23:39:00	23:55:12	02:18:36
Wake time (timestamp)	The estimated time a user gets out of bed after sleep	7:22:12	07:37:48	02:12:36
Sleep duration (h, min)	The aggregate time spent in bed (see Sleep measures)	6 h 54 min	7 h 42 min	12 min
Falling-asleep-duration (min)	The recorded time before a user falls asleep once in bed	6 min	6 min	28 min
Waking-up-duration (min)	The recorded time needed for a user to get out of bed after waking up	0 min	8 min	15 min
Mid-awake-duration (min)	The recorded time a user was awake while in bed	22 min	35 min	64 min

### Sleep measures

While sleep labs are the gold standard for obtaining objective sleep measurements in a clinical setting via advanced medical equipment like polysomnography and accelerometers, studies have shown that alternative, less expensive, and scalable solutions like using consumer-grade wearable devices can be a reliable way to study sleep^18,26^. The discussion of studies that compare wearable device measurement accuracy to gold standard actigraphy can be found in the Supplementary Material. In this study, sleep measures were collected using consumer-grade wrist-worn devices serviced by Nokia. All the measurements in our dataset were collected by the same version and type of wearable. These devices offer sleep-tracking functionality that continuously collects movement data through an accelerometer, aggregates it at a minute-level granularity, and uses it as input for a proprietary algorithm to estimate whether the person is sleeping. The above measurements in Table 2, logged in units of minutes, can be used to model conventional sleep metrics, including MSFsc^27^, hours overslept on free days or non-workdays^37^, the K-hour deviation^38^, and sleep efficiency^39^:

- **Sleep duration: **The aggregate time spent in bed, whether asleep or awake. This metric is often used as a proxy for the quantity of rest that people get at night^7,40,41^. The higher its value, the more time people sleep.
  $$Sleep\;duration = Wake\;time {-} Bedtime$$
- **Sleep history:** A seven-day average sleep duration that gives higher weights to recent days. It captures an aggregate measure of sleep behavior using the following formula, where *i* is the *i*th day in the past based on the target date. Note that sleep duration is weighted by a decaying exponential with a time constant of 7 days^42^, indicating that recent measurements have greater importance. The calculation of Sleep history is normalized such that weights sum to one, making the metric more interpretable as a weighted average of sleep duration over the past week^43^. The higher its value, the more time people slept over the past week.
  $$Sleep\;history = \frac{1}{{\mathop \sum \nolimits_{n = 1}^{7} e^{{ - \frac{n}{7}}} }} \mathop \sum \limits_{i = 1}^{7} e^{{ - \frac{i}{7}}} * sleep\;duration_{i}$$
- **Hours Overslept:** Additional hours of sleep on free days compared to weekdays. Hours overslept on free days can serve as a proxy for sleep debt, as it is a cumulative effect of not getting enough sleep^37^. When people need to wake up earlier than their normal biological time and subsequently oversleep on free days to compensate for the accumulated sleep debt during the week^44,45^. The higher its value, the more people sleep on free days.

$$Hours\;Overslept = SD_{f} - \left( {5*SD_{w} + 2*SD_{f} } \right)/7$$

,where $S{D}_{f}$ is the average sleep duration on free days per week and $S{D}_{w}$ is the average sleep duration on workdays^4^. In the current work, we set Sunday–Thursday as work nights and Friday–Saturday as free nights according to literature^4^.

- **MSFsc:** This metric is used to classify chronotypes, which refer to an individual's sleep pattern preference^27^. It is calculated by determining the midpoint of sleep on free days and adjusting it based on the individual's chronotype. To calculate the MSFsc, we first calculate Hours Overslept on free days which represent the number of extra hours an individual sleeps on her free days compared to her average sleep hours on workdays, as explained above. Once Hours Overslept is calculated, we then compute the MSFsc using the following formula^4^:
  $$MSF_{sc} = MSF - 0.5* Hours\;Overslept$$
  Here, MSF stands for the midpoint of sleep on free days, while SC represents the correction of sleep debt on workdays. The higher the value of MSFsc, the later an individual’s midpoint of sleep on free days becomes. In other words, a higher MSFsc value indicates a preference for staying up later and waking up later on free days.
- **K-hour deviation: **This metric is inspired by the concept of recommended sleep duration in society and is computed as the time deviation from k hours of duration in bed. This metric captures the extent to which people deviate from the recommended hours of rest (i.e., 7 or 8). The higher its value, the greater the divergence of people’s daily sleep time from the recommended sleep duration^38,46,47^. In this study, we provide the findings for the more typical k value of eight^48,49^ (see Supplementary Material for results with other k values).
  $$k{ - }hour\;deviation = \left| {Sleep\;duration - k} \right|$$
- **Efficiency:** The ratio of total hours slept over the total in-bed duration, as used in previous research^6,37,50^. This metric is used to represent sleep quality. Sleep efficiency can be measured by both surveys, as in Jenkins Sleep Problems Scale, or by sensors on wearable devices. The higher its value, the more quality sleep people acquire.
  $$Efficiency = \frac{ Hours\;Slept}{{In{ - }bed\;Duration}}$$

### Social constructs

To address the effect of culture on sleep quantity and quality, we examined how these measures varied according to popular social constructs. From Hofstede’s insights portal [Hofstede’s cultural dimensions: https://www.hofstede-insights.com/product/compare-countries], we obtained data in four dimensions for the 19 cities examined in the current study. Since some measures were skewed, we log-transformed these values if they were not normally distributed.

- The Individualism (IDV) dimension captures the degree to which a culture reinforces individual achievement and relationships. Greater values indicate more Individualism.
- The Uncertainty Avoidance (UAI) dimension captures the degree to which uncertain situations make members of a culture feel threatened. Greater values indicate a more regulated life.
- The Long-term Orientation (LTO) dimension captures the extent to which a culture believes in a stable society based on family and where various behaviors are expected. Greater values indicate long-term-oriented societies that value self-development.
- The Indulgence (IND) dimension captures the degree to which a society allows relatively free gratification of basic and natural human drivers related to enjoying life and having fun. The contrasting idea is restraint, which stands for a society that suppresses gratification of needs and regulates it by means of strict social norms.

We also considered the Gross domestic product (GDP) as a commonly used socio-economic factor that reflects the wealth of an area.

- log-GDP: For each city, we collected yearly GDP data from the OECD regional statistics, then averaged and log-transformed it using the natural logarithm due to its skewed distribution (log-GDP: min = 11.31, max = 14.38, median = 12.65, mean = 12.74, and SD = 0.95).

### Principal component analysis (PCA) for dimension reduction of sleep traits

For each user in our dataset, we computed mean values of five sleep metrics: sleep duration, sleep history, MSFsc, K-hour deviation, and sleep efficiency. When computing these sleep features, the number of target users decreased to 23,812 as we discarded user data if data points on free days and workdays fell below a threshold: every week in our data contained at least one free day and two workdays. While the five sleep measures reflected various sleep aspects, some captured similar concepts. With the sleep records of thousands of users at hand, we could assess which fundamental dimensions captured different aspects of sleep patterns in a data-driven fashion. We did so by running a principal component analysis (PCA) to reduce the dimensionality of our sleep predictors (see Table S5, which lists the PCA loading matrix). We found that the first two principal components had eigenvalues larger than one (2.98 and 1.18, respectively) and, as depicted in Fig. S3(B), accounted for 83.14% of the total variance, which is high, considering that 60% variance is typically deemed satisfactory in social sciences^51^. The first principal component captured aspects of sleep related to quantity (i.e., how much the user slept). The second component was related to sleep quality features (i.e., how well the user slept). Thus, we named these two dimensions Sleep Quantity and Sleep Quality, respectively (see Text S2 for more details).

### Linear regressions

To examine the correlation between sleep dimensions and popular social constructs at a city-specific level, linear regression models were used after identifying two sleep dimensions using principal component analysis (PCA). Six linear regression models (M1 to M6) were used in this study. The baseline models (M1 and M4) included only log-GDP and controlled for the median age of users. The full models (M2 and M5) incorporated all social constructs, while the final compact models (M3 and M6) contained only significant factors selected through the StepAIC feature selection method. The StepAIC is a feature selection method used in linear regression analysis [https://www.rdocumentation.org/packages/MASS/versions/7.3-58.3/topics/stepAIC]. It is based on the Akaike Information Criterion (AIC), which quantifies the goodness of fit of a model while penalizing the number of variables included. StepAIC is a backward selection method that starts with the full model and iteratively removes the variable with the least significant contribution until a model with the best AIC value is obtained. This method helps to simplify the model by selecting only the most significant variables while avoiding overfitting. The goal of these models was to explain the effect of key societal factors on sleep measures.

### Propensity score matching (PSM) analysis

PSM is a method used to test the causal effect by identifying pairs of treatment and control units that are matched based on their propensity scores^52^. In our case, we used PSM to quantify the effect of physical activity on two sleep dimensions: sleep quantity and sleep quality, at an aggregated user level. We chose average daily steps as a proxy to gauge the degree of daily activity.

The propensity scores are the conditional probabilities of being assigned to the treatment group, given some vector of covariates. In our analysis, the treatment group consisted of users who were in the top one-third of their country in terms of their average daily steps (i.e., more active users). Accordingly, the control group consiseds of the bottom one-third of users (i.e., less active users). Three covariates were used for the propensity score calculations: age, BMI, and wearable data recording ratio. [Wearable data recording ratio was calculated as the total number of daily records per period of using a wearable device in days. This measure addresses a number of missing data points as well as the persistence of the users in using the wearables.] The propensity scores were calculated using logistic regression with Lasso regularization (ƛ = 0.001).

In the next step, for each unit in the treatment group, we matched the five (as a hyperparameter) most similar units from the control group based on the propensity scores by the K-Nearest Neighbor algorithm. The similarity of neighbors was calculated using Mahalanobis distance, which normalizes the distance between two points in a multivariate space. To measure the effect of activity on the sleep dimensions, we calculated the estimated average treatment effect (EATE) by the following formula^53^, where $T$ is the treatment group, ${M}_{t}$ is the corresponding matched set from the control group, and y is the desired sleep dimension (PCA coordinates of sleep quantity or sleep quality). ${N}_{t}$ is the number of treatment units, and ${N}_{{M}_{t}}$ is the size of matched control sets (in our case, ${N}_{{M}_{t}}$= 5):

$$EATE = \mathop \sum \limits_{t}^{T} \mathop \sum \limits_{m}^{{M_{t} }} \left( {\frac{{y_{t} - y_{m} }}{{N_{{M_{t} }} }}} \right)/N_{T}$$

## Supplementary Information

Supplementary Information.

## Data Availability

The data was collected at a large scale from Nokia wearable devices in the wild between 2014 and 2017. Consent was obtained from the users to access their anonymized records. Upon request, the corresponding authors can provide the aggregate city-level sleep metrics.
