# Supplementary material for: Social dimensions impact individual sleep quantity and quality
Source: Sci Rep. 2023 Jun 15;13:9681. doi: 10.1038/s41598-023-36762-5 (PMC10272146; doi:10.1038/s41598-023-36762-5)
Supplement: Supplementary file 1 — Supplementary Information. [file 41598_2023_36762_MOESM1_ESM.docx]

**Title**

Social dimensions impact individual sleep quantity and quality

**Authors**

Sungkyu Park^1,2^, Assem Zhunis^3,2^, Marios Constantinides^4^, Luca Maria Aiello^5,4^, Daniele Quercia^†4,6^, Meeyoung Cha^†2,3^

**Affiliations**

^1^Department of AI Convergence, Kangwon National University; Chuncheon, 24341, Republic of Korea.

^2^Data Science Group, Institute for Basic Science; Daejeon, 34126, Republic of Korea.

^3^School of Computing, KAIST; Daejeon, 34141, Republic of Korea.

^4^Nokia Bell Labs, Cambridge CB3 0FA, United Kingdom.

^5^IT University, Copenhagen, Denmark.

^6^Centre for Urban Science and Progress, King’s College London, London, UK.

Sungkyu Park and Assem Zhunis​ equally contributed to this work

^†^Corresponding authors. Email:daniele.quercia@nokia-bell-labs.com; mcha@ibs.re.kr

**This PDF file includes:**

Supplementary Text S1 to S5

Figs. S1 to S4

Tables S1 to S8

**Supplementary Text**

**Text S1: Sleep patterns by age and gender**

Age and gender have been identified as key dominating individual factors of sleep. For Fig S1, we first calculated the average values for each user and then averaged the values by age, i.e., an average of an average. We could see that the relatively young and old generations tend to sleep more than the middle-aged group, as shown in Fig S1(A). This may be because middle-aged people have more regulated lifestyle patterns. Based on Fig S1(B), we also showed that there is no statistical difference between Workdays and Freedays on Sleep duration in our dataset (Mann-Whitney U-test, U=11.5, p=0.46 with 10mins bin), which is contrary to one reference (3), and that is the reason why we have not included Social jetlag-related features, which can be derived by Sleep midpoint on Freedays - Sleep midpoint on Workdays. Meanwhile, we found that female users tend to sleep more than male users as a baseline across ages, as depicted in Fig S1(C).

**Text S2: Principal component analysis to reduce the dimension of sleep traits**

The first component explains 59.52% of the variance. Three sleep metrics were loaded in that component (PC1): log Bed-duration, Sleep history, and MSFsc; these metrics capture aspects of sleep related to quantity, we named PC1 as Sleep Quantity. The second component explains 23.62% of the variance. Two sleep metrics were loaded in that component (PC2): K-Hour Deviation (k=8) and Sleep efficiency; as these two metrics capture aspects of sleep-related to quality, we named PC2 as Sleep Quality. Table S5 shows the most correlated sleep metrics with each PCA component for different k values for the K-hour deviation metric.

**Text S3: Handling outliers**

In (5), the authors removed data that reported too early or too late sleep, as described below in italics. We have further applied the same filtering process, and the results are similar to our original dataset - the directions are the same, whereas the magnitudes become slightly diminished. We speculate that the effect of our models becomes weaker with filtering because we exclude some data points showing drastic sleep traits compared to the ones with a normal range of sleep traits. Thus, in this paper, we included results obtained from the original dataset analysis.

*Outliers were removed from the data set by applying the following exclusion criteria. Users were excluded if the difference between their wake times, bedtimes, or sleep duration and those quantities’ respective peaks of 07:00 local time, 23:00 local time, and 8 hours of sleep exceeded 4 hours in magnitude. Hence, users reporting wake times strictly before 0300 or after 1100 were excluded, as well as users reporting bedtimes strictly before 1900 or after 0300 and sleep durations strictly less than 4 hours or greater than 12 hours. In addition, users strictly under the age of 18 or above the age of 85 were also excluded. It should be noted that most shift workers are likely to be excluded from our analysis. After this outlier's removal, 5450 users remained in the data set.*

**Text S4: Comparison Data between the Most active and the Least Active Users**

Before going through a rigorous test with the propensity score matching analysis, we check the group difference between the top 30% and bottom 30% of users in daily steps in Table S2. We compare two groups on Bedtime, Waketime, and Sleep duration. In general, the top 30% of users tend to go to bed and wake up earlier and sleep less across cities. It is intriguing to note that more exercise is associated with less sleep duration, and it may also resonate that sleep quantity and quality lie in different spaces.

**Text S5: Comparison Data between Surveys and Wearables**

There could be two possible biases explaining the discrepancy in Fig 4: sample bias and reporting bias. One could speculate that specific demographics, such as age and profession, influence sleep traits and cause sample bias. In order to stratify the analysis by ruling out these biases as much as possible, we compare again by controlling age and profession on the given datasets in Table S3. Meanwhile, as the data from (5) and ours both cover a broad range of age bands (from young adults to seniors) across multiple countries and their sample users may have similar tech-friendly characteristics, we can posit that the discrepancy is mainly induced by reporting bias, not by sample bias.

**Text S6: Comparison of Wearable Devices to Gold Standard Methods**

Wrist-worn sleep trackers offer several advantages as personal sleep monitoring devices. They provide a convenient and non-intrusive option for tracking sleep patterns without the need for a controlled laboratory environment. Wrist trackers are easily wearable and portable, allowing for continuous monitoring over extended periods in natural settings. They provide users with valuable insights into their sleep duration, sleep quality, and trends over time. While wearable trackers may not achieve the same level of accuracy as gold-standard methods like polysomnography (PSG), studies have demonstrated their potential for sleep research [1-5]. For instance, compared to smartphone accelerometer applications there were no significant differences in sleep parameters like TST, WASO, or SE between PSG and actigraphy or wristband trackers [1]. Research also concluded that wearables have promising clinical utility in children with sleep-disordered breathing [1]. Roberts et al. (2020) discovered strong correlations between data from consumer wearables and reference devices, allowing for the development of sleep-wake models [2]. Similarly, Lee et al. (2019) concluded that consumer-grade wearable devices can measure sleep duration comparable to research-grade actigraphy, with appropriate threshold adjustments for sleep staging [3]. It was also found that for the younger population, wearables achieved even higher agreement [4]. These findings highlight the growing body of research comparing wearable devices with gold-standard methods in sleep tracking. While wearables may not fully replace PSG, they can still be valuable tools for observational research and delivering behavioral interventions [5].

1. Toon, E., Davey, M. J., Hollis, S. L., Nixon, G. M., Horne, R. S., & Biggs, S. N. (2016). Comparison of commercial wrist-based and smartphone accelerometers, actigraphy, and PSG in a clinical cohort of children and adolescents. Journal of Clinical Sleep Medicine, 12(3), 343-350.
2. Roberts, D. M., Schade, M. M., Mathew, G. M., Gartenberg, D., & Buxton, O. M. (2020). Detecting sleep using heart rate and motion data from multisensor consumer-grade wearables, relative to wrist actigraphy and polysomnography. Sleep, 43(7), zsaa045.
3. Lee, X. K., Chee, N. I., Ong, J. L., Teo, T. B., van Rijn, E., Lo, J. C., & Chee, M. W. (2019). Validation of a consumer sleep wearable device with actigraphy and polysomnography in adolescents across sleep opportunity manipulations. Journal of Clinical Sleep Medicine, 15(9), 1337-1346.
4. Wulterkens, B.M., Fonseca, P., Hermans, L.W., Ross, M., Cerny, A., Anderer, P., Long, X., van Dijk, J.P., Vandenbussche, N., Pillen, S. and van Gilst, M.M., 2021. It is all in the wrist: wearable sleep staging in a clinical population versus reference polysomnography. Nature and Science of Sleep, pp.885-897.
5. Baron, K. G., Duffecy, J., Berendsen, M. A., Mason, I. C., Lattie, E. G., & Manalo, N. C. (2018). Feeling validated yet? A scoping review of the use of consumer-targeted wearable and mobile technology to measure and improve sleep. Sleep medicine reviews, 40, 151-159.

**A                B                                                   C**


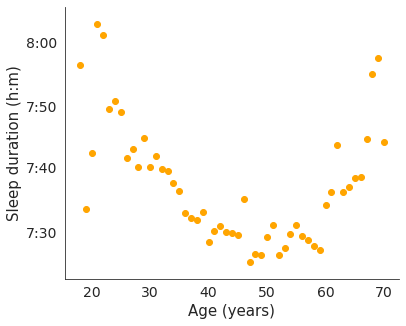

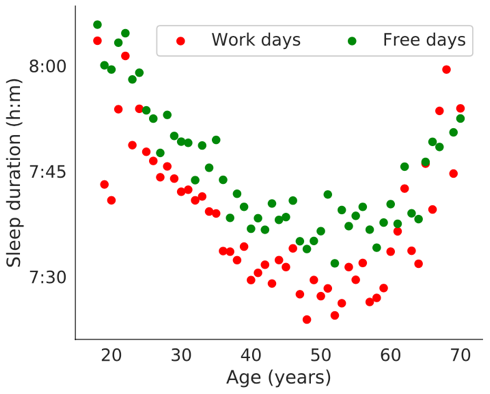
 
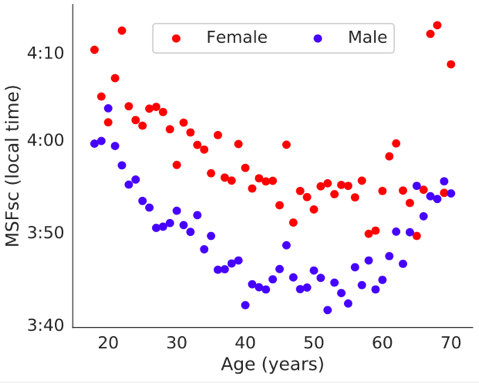


**Fig. S1.**

**(A)** Distribution of sleep duration by age. **(B)** Distribution difference of sleep duration between workdays and non-workdays across age groups. **(C)** Distribution difference of sleep duration between workdays and non-workdays across gender.


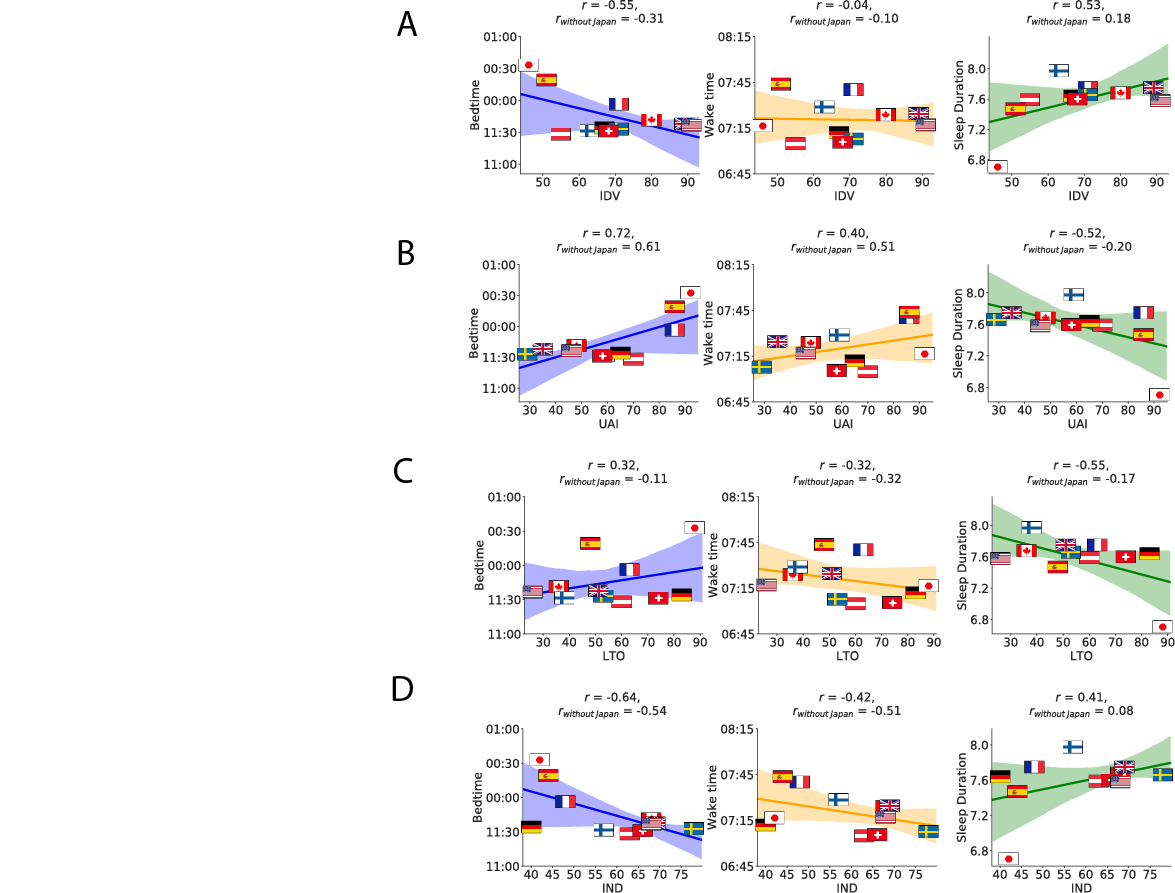


**Fig. S2. Cultural dimensions and sleep traits**

Linear regressions and correlations between various cultural dimensions and sleep traits, including median Bedin and Bedout time and Sleep duration. **(A)** IDV (individualism). **(B)** UAI (uncertainty avoidance index). **(C)** LTO (long-term orientation). **(D)** IND (indulgence).

**A**

**
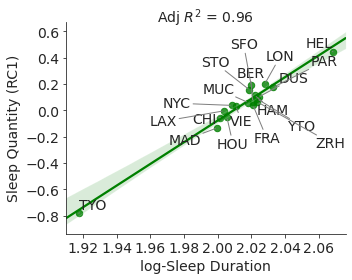
    
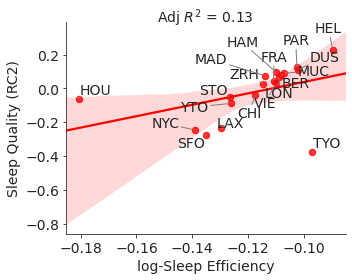
**

**B**

**
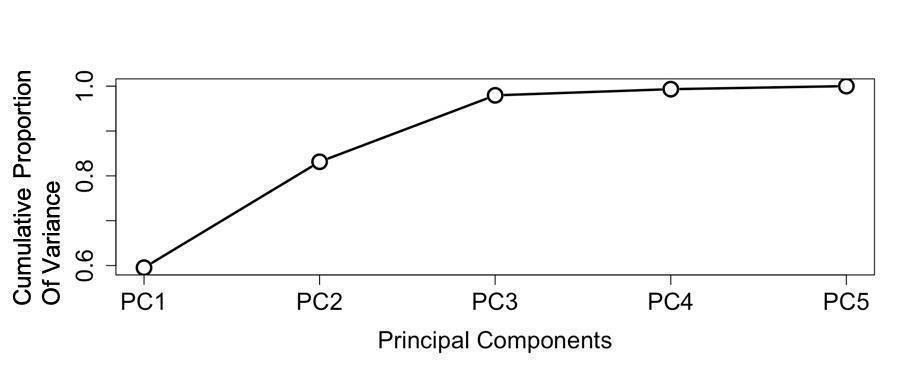
**

**Fig. S3. Sleep metrics**

**(A)** Correlations of the first and second principal components with log-Sleep Duration and log-Sleep Efficiency, respectively. **(B)** Cumulative variance explanation on PCA. The first component explains 60% of the variance (PC1), the second explains 24% (PC2), the third explains 15% (PC3), the fourth explains 1.4% (PC4), and the fifth explains 0.7% (PC5).

**A**


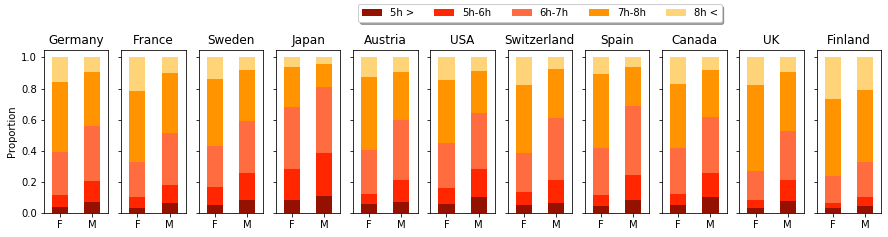


**B**


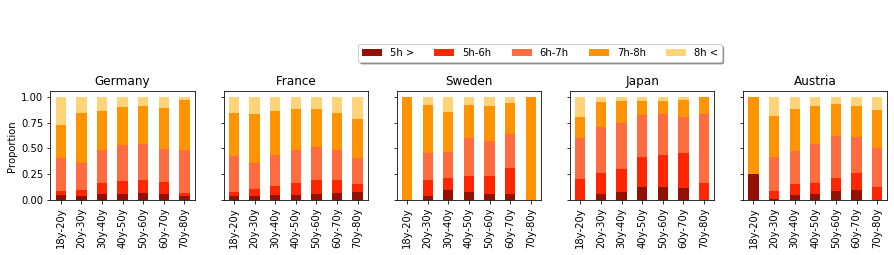

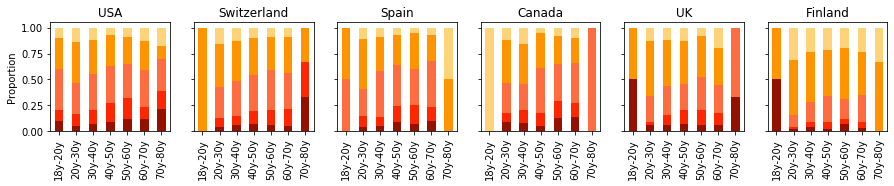


**Fig. S4. Sleep duration across gender and age groups**

**(A)** Proportion of female (F) and male (M) users sleeping on average less than 5, 5 to 6, 7 to 8, and more than 8 hours per night. **(B)** Proportion of users across seven age groups sleeping on average less than 5, 5 to 6, 7 to 8, and more than 8 hours per night

| **Country** | **Bedtime** | **Wake Time** | **Sleep duration** | **Sleep efficiency** |
| --- | --- | --- | --- | --- |
| Finland | 11:43 PM | 7:43 AM | 8h 01m | 92.76% |
| France | 12:06 AM | 7:53 AM | 7h 45m | 92.41% |
| UK | 11:52 PM | 7:38 AM | 7h 44m | 93.61% |
| Sweden | 11:48 PM | 7:26 AM | 7h 41m | 92.79% |
| Germany | 11:45 PM | 7:25 AM | 7h 39m | 92.57% |
| Switzerland | 11:39 PM | 7:20 AM | 7h 39m | 92.11% |
| Canada | 11:55 PM | 7:40 AM | 7h 38m | 92.93% |
| Austria | 11:38 PM | 7:20 AM | 7h 35m | 92.52% |
| US | 11:51 PM | 7:28 AM | 7h 34m | 93.4% |
| Spain | 12:30 AM | 8:01 AM | 7h 28m | 92.87% |
| Japan | 12:42 AM | 7:38 AM | 6h 51m | 93.1% |

**Table S1.**

Average of median bedtime, wake time, sleep duration, and sleep efficiency by countries, sorted in descending order by sleep duration.

| **City** | **Steps (count)** | | | **Bedtime (timestamp)** | | | **Wake time (timestamp)** | | | **Sleep duration (h m)** | | |
| --- | --- | --- | --- | --- | --- | --- | --- | --- | --- | --- | --- | --- |
|  | **Bottom 30%** | **Top**  **30%** | **All** | **Bottom 30%** | **Top**  **30%** | **All** | **Bottom 30%** | **Top**  **30%** | **All** | **Bottom 30%** | **Top**  **30%** | **All** |
| **Berlin** | 4,047 | 10,146 | 7,156 | 0:01 | 0:00 | 23:56 | 7:44 | 7:27 | 7:34 | 7h 43m | 7h 26m | 7h 37m |
| **Chicago** | 3,178 | 9,450 | 6,446 | 0:06 | 0:04 | 0:07 | 7:49 | 7:29 | 7:38 | 7h 42m | 7h 24m | 7h 31m |
| **Dusseldorf** | 4,100 | 10,137 | 7,127 | 0:14 | 23:55 | 0:01 | 7:58 | 7:30 | 7:40 | 7h 43m | 7h 35m | 7h 39m |
| **Frankfurt am Main** | 3,657 | 9,703 | 6,808 | 23:46 | 23:56 | 23:56 | 7:32 | 7:31 | 7:34 | 7h 46m | 7h 34m | 7h 38m |
| **Hamburg** | 3,820 | 10,045 | 6,972 | 0:09 | 23:45 | 23:58 | 7:53 | 7:19 | 7:37 | 7h 44m | 7h 33m | 7h 38m |
| **Helsinki** | 4,135 | 9,765 | 6,949 | 0:11 | 23:48 | 23:58 | 8:14 | 7:41 | 7:57 | 8h 03m | 7h 53m | 7h 59m |
| **Houston** | 2,823 | 8,845 | 5,930 | 0:12 | 23:48 | 23:59 | 7:54 | 7:09 | 7:30 | 7h 42m | 7h 20m | 7h 30m |
| **London** | 4,069 | 10,623 | 7,508 | 0:13 | 0:02 | 0:08 | 7:56 | 7:40 | 7:49 | 7h 43m | 7h 37m | 7h 41m |
| **Los Angeles** | 3,174 | 9,625 | 6,461 | 0:14 | 0:09 | 0:10 | 7:55 | 7:32 | 7:41 | 7h 41m | 7h 22m | 7h 31m |
| **Madrid** | 4,327 | 10,371 | 7,400 | 0:40 | 0:38 | 0:41 | 8:19 | 7:57 | 8:10 | 7h 38m | 7h 18m | 7h 29m |
| **Munich** | 3,919 | 9,684 | 6,917 | 0:03 | 23:49 | 23:56 | 7:35 | 7:25 | 7:33 | 7h 31m | 7h 35m | 7h 36m |
| **New York** | 3,517 | 10,275 | 7,163 | 0:19 | 0:19 | 0:17 | 8:08 | 7:42 | 7:51 | 7h 48m | 7h 22m | 7h 34m |
| **Paris** | 4,061 | 10,055 | 7,164 | 0:21 | 0:23 | 0:20 | 8:12 | 7:58 | 8:04 | 7h 51m | 7h 35m | 7h 43m |
| **San Francisco** | 3,762 | 9,932 | 7,145 | 0:31 | 0:09 | 0:20 | 8:13 | 7:41 | 7:59 | 7h 41m | 7h 31m | 7h 39m |
| **Stockholm** | 4,363 | 10,287 | 7,549 | 0:03 | 23:54 | 0:00 | 7:40 | 7:27 | 7:37 | 7h 37m | 7h 33m | 7h 36m |
| **Tokyo** | 4,446 | 10,422 | 7,483 | 1:26 | 0:44 | 1:02 | 8:31 | 7:28 | 7:56 | 7h 04m | 6h 44m | 6h 54m |
| **Toronto** | 3,435 | 9,701 | 6,719 | 0:27 | 0:07 | 0:13 | 8:19 | 7:40 | 7:54 | 7h 51m | 7h 33m | 7h 40m |
| **Vienna** | 3,574 | 9,696 | 6,774 | 0:04 | 23:56 | 23:59 | 7:34 | 7:30 | 7:32 | 7h 29m | 7h 33m | 7h 33m |
| **Zurich** | 3,743 | 9,927 | 7,030 | 23:55 | 23:47 | 23:52 | 7:43 | 7:19 | 7:31 | 7h 48m | 7h 32m | 7h 38m |

**Table S2.**

Group difference between top and bottom 30% of users on Bedtime, Wake time, and Sleep duration. Users were grouped based on daily Steps counts.

| **Dataset** | **Data type** | **Age band** | **# Samples** | **User base** |
| --- | --- | --- | --- | --- |
| Walch et al. (5) | Survey | 18-85 | 8,070 | Mobile jet-lag application users from 20 countries |
| Soldatos et al. (43) | Survey | 15–99 | 35,327 | Survey participants from 10 countries |
| Zhang et al. (4) | Wearables | 10-70 | 71,176 | Wearable users in China |
| Park et al. (24) | Wearables | 18-28 | 42 | Recruited students in South Korea |
| Ong et al. (52) | Wearables | 15-80 | 23,680 | Wearable users from five countries |
| Ours | Wearables | 18-80 | 30,179 | Wearable users from 11 countries |

**Table S3.**

Basic statistics of the multiple datasets retrieved by surveys or wearable devices.

| **Variable** | **Distribution** | **Min** | **Max** | **Median** | **Mean** | **SD** |
| --- | --- | --- | --- | --- | --- | --- |
| log-sleep duration | 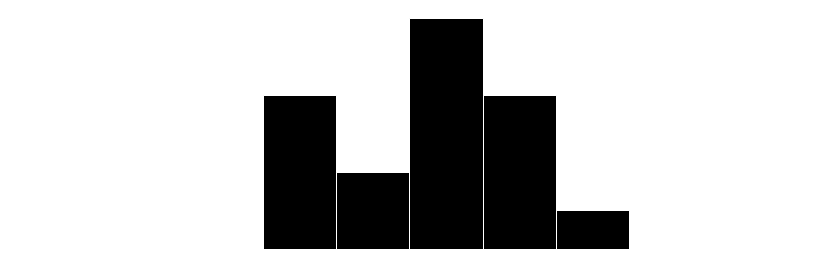 | 1.92 | 2.07 | 2.02 | 2.01 | 0.03 |
| log-K-hour rule (k=8) | 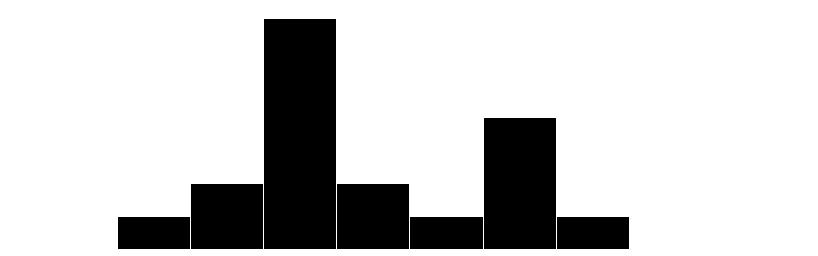 | 0.25 | 0.62 | 0.30 | 0.33 | 0.08 |
| log-Sleep Efficiency | 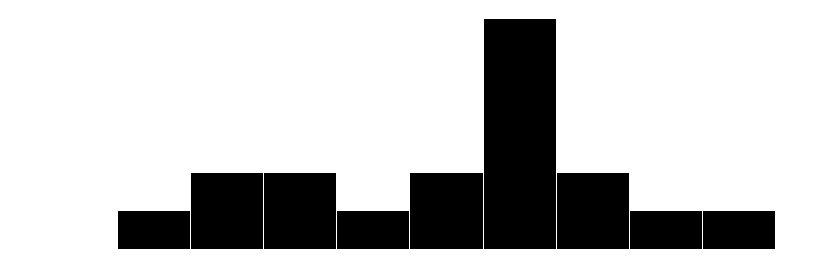 | -1.81 | -0.09 | -0.11 | -0.12 | 0.02 |
| Hours Overslept | 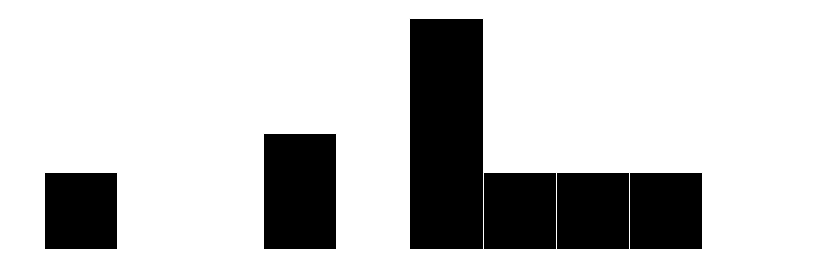 | 3.39 | 0.13 | 0.08 | 0.08 | 0.03 |
| SJL | 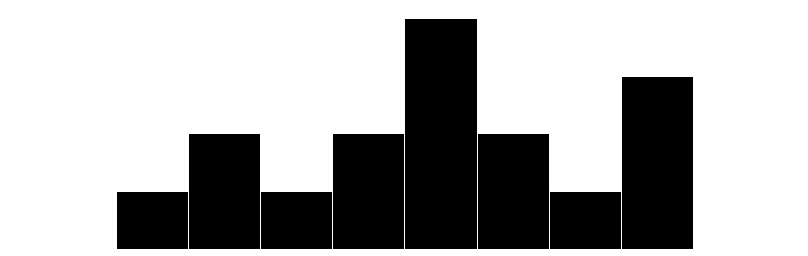 | 4.99 | 0.10 | 0.06 | 0.06 | 0.02 |
| Sleep History | 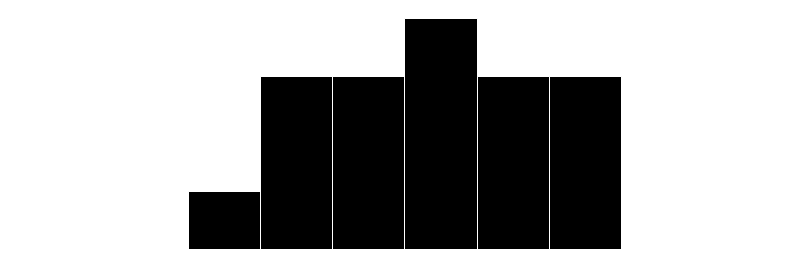 | 7.03 | 8.11 | 7.74 | 7.72 | 0.20 |
| MSFsc | 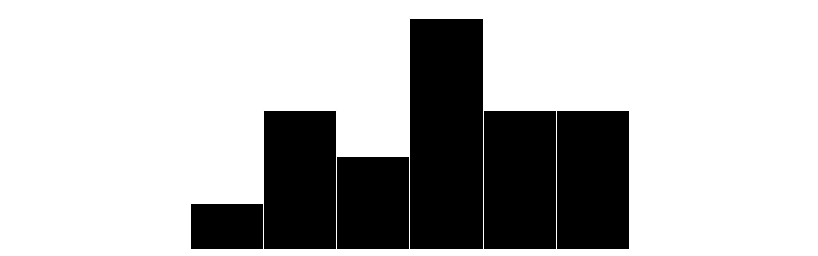 | 3.50 | 4.05 | 3.86 | 3.85 | 0.10 |

**Table S4.**

Frequency distributions and statistics of sleep metrics aggregated by cities.

| **k-value** | **6** | | **7** | | **8** | | **9** | | **Trait** |
| --- | --- | --- | --- | --- | --- | --- | --- | --- | --- |
| **Sleep metric** | **PC1** | **PC2** | **PC1** | **PC2** | **PC1** | **PC2** | **PC1** | **PC2** |  |
| *log-Sleep Duration* | **0.97** | 0.00 | **0.96** | -0.05 | **0.98** | 0.09 | **0.98** | -0.06 | Sleep quantity |
| *Sleep History* | **0.97** | -0.01 | **0.97** | -0.09 | **0.97** | 0.02 | **0.95** | -0.09 | Sleep quantity |
| *MSFsc* | **0.98** | -0.02 | **0.98** | -0.09 | **0.98** | 0.03 | **0.97** | -0.09 | Sleep quantity |
| *log-K-hour Deviation* | **0.91** | -0.10 | 0.50 | **-0.52** | -0.26 | **-0.73** | **-0.78** | -0.24 | Sleep quality |
| *log-Sleep Efficiency* | -0.06 | **1.00** | 0.05 | **0.93** | -0.16 | **0.82** | -0.02 | **0.98** | Sleep quality |
| **Eigenvalue** | 3.69 | 0.99 | 3.17 | 1.06 | 2.98 | 1.18 | 3.41 | 1.04 | — |
| **Explained variance** | 0.73 | 0.20 | 0.62 | 0.23 | 0.60 | 0.24 | 0.68 | 0.21 | — |
| **Subscale Cronbach’s** $\alpha$ | 0.82 | — | 0.76 | -0.25* | 0.76 | -0.22* | 0.39 | — | — |

**Table S5.**

Composite metrics of sleep identified by PCA after varimax rotation on the values of sleep metrics at individual-level (N = 23,812). *Negative α has been reported due to the non-linearity nature of the *K*-hour Deviation rule by definition, i.e., |Sleep duration - k hours|.

| **Age group** | **Bedtime** | | **Wake time** | | **Sleep Duration** | |
| --- | --- | --- | --- | --- | --- | --- |
| 18-20 | 2.45* | | 2.05* | | -1.78̇ | |
| 20-30 | 8.98*** | | 5.00*** | | -8.17*** | |
| 30-40 | 12.16*** | | 5.76*** | | -11.91*** | |
| 40-50 | 7.96*** | | 1.43 | | -10.92*** | |
| 50-60 | 2.68** | | -2.38* | | -7.04*** | |
| 60-70 | -2.47* | | -4.97*** | | -2.62** | |
| 70-80 | 0.89 | | -1.31 | | -2.91** | |

**Table S6.**

Results of the t-test for gender differences in bedtime, waketime, and sleep duration across age groups. Statistical significances are marked with a number of *’s based on their significance levels (i.e., ***p< 0:01; **p< 0:05; *p< 0:1).

| **Feature** | **Sleep Quantity (RC1)** | **Sleep Quality (RC2)** |
| --- | --- | --- |
| Canada | 0.06 | -0.12** |
| Finland | 0.41*** | 0.20*** |
| France | 0.14*** | 0.09** |
| Germany | 0.04 | 0.05 |
| Japan | -0.81*** | -0.41*** |
| Spain | -0.16*** | 0.04 |
| Sweden | 0.12** | -0.08 |
| Switzerland | 0.03 | -0.00 |
| United Kingdom | 0.17*** | 0.04 |
| United States | -0.01 | -0.21*** |
| *Intercept* | 0.03 | 0.03 |
| ***Adjusted R²*** | **0.100** | **0.031** |

**Table S7.**

Results of the regression models exploring the impact of users' location (which country users live in), represented as dummy variables, on individual-level analysis of sleep quantity (RC1) and quality (RC2). Statistical significances of model coefficients are marked with a number of *’s based on their significance levels (i.e., ***p< 0:01; **p< 0:05; *p< 0:1).

| **Country** | **Change in Sleep Duration**  **(min)** | **MAE**  **(min)** | **Change in K-hour Deviation with k=8**  **(min)** | **MAE**  **(min)** |
| --- | --- | --- | --- | --- |
| Austria | 0.75 | 48.07 | -0.84 | 30.41 |
| Canada | -0.27 | 48.09 | -1.39 | 31.79 |
| Germany | -2.14 | 44.84 | -0.93 | 28.37 |
| Spain | -1.93 | 42.40 | -1.02 | 25.13 |
| Sweden | -4.04 | 41.75 | -1.02 | 25.13 |
| Switzerland | -0.32 | 43.80 | -1.25 | 28.30 |
| France | -2.97 | 44.26 | -0.88 | 29.10 |
| United States | -2.47 | 52.03 | -1.28 | 35.66 |
| United Kingdom | -2.72 | 44.61 | -1.23 | 44.30 |
| Japan | -5.11 | 52.70 | -2.34 | 37.41 |
| Finland | -4.17 | 44.55 | -3.69 | 30.04 |

**Table S8.**

The regression model coefficients examining the relationship between increasing average daily 1000 steps per day at the city level and changes in sleep duration and K-hour deviation.
